# The Role of Microbiome and Genotype in *Daphnia magna* upon Parasite Re-Exposure

**DOI:** 10.3390/genes12010070

**Published:** 2021-01-07

**Authors:** Lore Bulteel, Shira Houwenhuyse, Steven A. J. Declerck, Ellen Decaestecker

**Affiliations:** 1 Laboratory of Aquatic Biology, Department of Biology, University of Leuven-Campus Kulak, E. Sabbelaan 53, 8500 Kortrijk, Belgium; shira.houwenhuyse@kuleuven.be (S.H.); ellen.decaestecker@kuleuven.be (E.D.); 2 Netherlands Institute of Ecology (NIOO-KNAW), Droevendaalsesteeg 10, 6700 AB Wageningen, The Netherlands; S.Declerck@nioo.knaw.nl; 3 Laboratory of Aquatic Ecology, Evolution and Conservation, Department of Biology, KULeuven, 3000 Leuven, Belgium

**Keywords:** *Daphnia magna*, diversity, dysbiosis, genotype, gut microbiome, parasite re-exposure, tolerance

## Abstract

Recently, it has been shown that the community of gut microorganisms plays a crucial role in host performance with respect to parasite tolerance. Knowledge, however, is lacking on the role of the gut microbiome in mediating host tolerance after parasite re-exposure, especially considering multiple parasite infections. We here aimed to fill this knowledge gap by studying the role of the gut microbiome on tolerance in *Daphnia magna* upon multiple parasite species re-exposure. Additionally, we investigated the role of the host genotype in the interaction between the gut microbiome and the host phenotypic performance. A microbiome transplant experiment was performed in which three germ-free *D. magna* genotypes were exposed to a gut microbial inoculum and a parasite community treatment. The gut microbiome inocula were pre-exposed to the same parasite communities or a control treatment. *Daphnia* performance was monitored, and amplicon sequencing was performed to characterize the gut microbial community. Our experimental results showed that the gut microbiome plays no role in *Daphnia* tolerance upon parasite re-exposure. We did, however, find a main effect of the gut microbiome on *Daphnia* body size reflecting parasite specific responses. Our results also showed that it is rather the *Daphnia* genotype, and not the gut microbiome, that affected parasite-induced host mortality. Additionally, we found a role of the genotype in structuring the gut microbial community, both in alpha diversity as in the microbial composition.

## 1. Introduction

Recently, it has been shown that the microbial community is involved in multiple processes in host biology, such as food digestion, metabolic regulation, developmental signaling, behavior, and social interactions [1,2,3]. A part of these microbiota resides in the gut, where they are in direct and continuous contact with host tissues [4,5]. These gut symbionts, composed of bacteria, archaea, anaerobic fungi, protozoa, and viruses, provide nutrients, detoxify toxins, and contribute to the host’s development and growth [2,6,7,8]. The gut microbiome can also provide protection against parasites [9,10,11]. There is growing recognition that the effects of the gut microbiota and parasites on the host are intertwined. Infection with parasites, e.g., intestinal helminths, can significantly disrupt or restructure the host’s microbial community, both in invertebrates [12,13,14] and in vertebrates [15,16].

The gut microbiome can shape and enhance the host’s immune system by up-regulation of mucosal activity and induction of antimicrobial peptides [17,18,19]. Additionally, a stable and diverse gut microbiome can prevent colonization and limit detrimental effects of invading parasites, which is generally associated with interactions between the gut microbiome and the immune system [20,21]. An unbalanced or maladapted microbiome, i.e., dysbiosis of the microbial community, may result in a microbial community which can be both in low-diversity and modified metabolic state [22]. This unbalanced microbiome can increase susceptibility to parasites [23], and is set in motion by a number of direct or indirect mechanisms in the gut [24], e.g., by subverting the immune system, leading to further negative effects [25]. A lower microbial diversity has also been correlated with either a higher abundance of, or an increased susceptibility to, low abundant, opportunistic parasites [26,27,28]. Even though studies mostly focus on the beneficial effects of the gut microbiota, particular commensal gut communities can also increase host susceptibility to disease [29], and even turn harmful under certain conditions (i.e., pathobiont) [22,30]. 

The reciprocal role between parasites and the host’s microbiome has been studied in multiple invertebrates (beetles [14], bumblebees [9,11,31], *C. elegans* [32], fruit flies [33], oysters [34]). The honey bees are an especially well-studied group in that perspective; a protective function of the gut microbiome against parasites has been shown through controlled microbial inoculations [35,36]. However, only one study so far has been undertaken on the experimental model system, the water flea *Daphnia* and its parasites [37]. Sison-Mangus et al. [38] found no evidence that a host’s microbiota regulated resistance against the bacterial parasite *Pasteuria ramosa*, but did find significant differences between microbial communities depending on the host’s genotype. *Daphnia* studies also showed clear genotype-specific responses of the gut microbiome, mainly upon toxic cyanobacterial exposure in *Daphnia* tolerance [39,40,41] or exposure to environmental microbial pools [42,43].

Studies have been undertaken on the reciprocal role between the host microbiome and parasites. No studies, however, have looked into the mediating capacity of the microbiome in parasite tolerance after re-exposure to that same parasite community. Filling this knowledge gap must be one of the next priorities in studying the complex interplay between the gut microbiome and parasite infection, especially because organisms are often infected by multiple parasites. To address this knowledge gap, we set up an experiment to investigate the reciprocal role of the gut microbial community and parasite exposure in host–multiple parasite interactions in *Daphnia magna* after parasite re-exposure. We first performed a microbiome adaptation experiment in which we exposed *Daphnia* populations to two parasite communities and one control treatment, assuming the host gut microbiomes will adapt to the parasite communities. Additionally, we included three *Daphnia* genotypes in our experimental design to reveal possible intraspecific responses in *Daphnia*-microbiome-multiple parasite interactions, which could induce microbiome mediated evolutionary responses linked to particular genotypes [2]. The gut microbial community can drive eco-evolutionary dynamics through its host by impacting life history traits. Combined with the important role of parasites and the genotype in regulating and shaping individual responses and host populations, such population effects can mediate changes up to community level and the whole ecosystem.

## 2. Materials and Methods 

### 2.1. Microbiome Adaptation Experiment 

**Figure 1:**
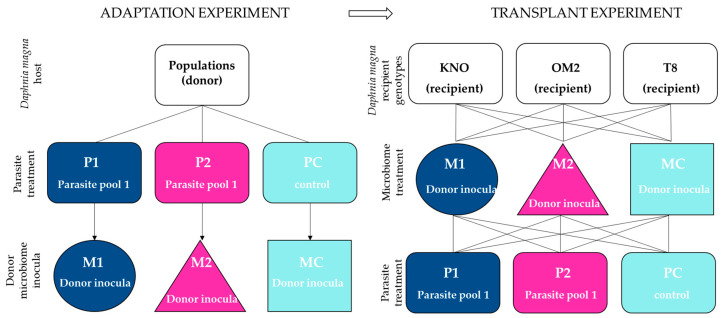
Schematic representation of the experimental design. In the microbial adaptation experiment (**left panel**), all donor populations received a parasite treatment (P1, P2, PC), after which the donor microbial inocula were obtained (M1, M2, MC) for the transplant experiment (**right panel**). P1 consisted of a pool of White Fat Cell Disease (WFCD) and *Binucleata daphniae.* P2 consisted of a pool of *Pasteuria ramosa, Ordospora colligata* and *Mitosporidium daphniae.* PC is the control treatment and consisted of a pool of healthy squashed *Daphnia* individuals. In the transplant experiment (**right panel**), sterile recipient individuals of three *Daphnia* genotypes (KNO, OM2, T8) were exposed to the donor microbiomes obtained from the adaptation experiment (M1, M2, or MC). Each of these microbiome treatments were crossed with the three parasite treatments (P1, P2, or PC; similar as in the microbiome adaptation experiment).

**Figure 2:**
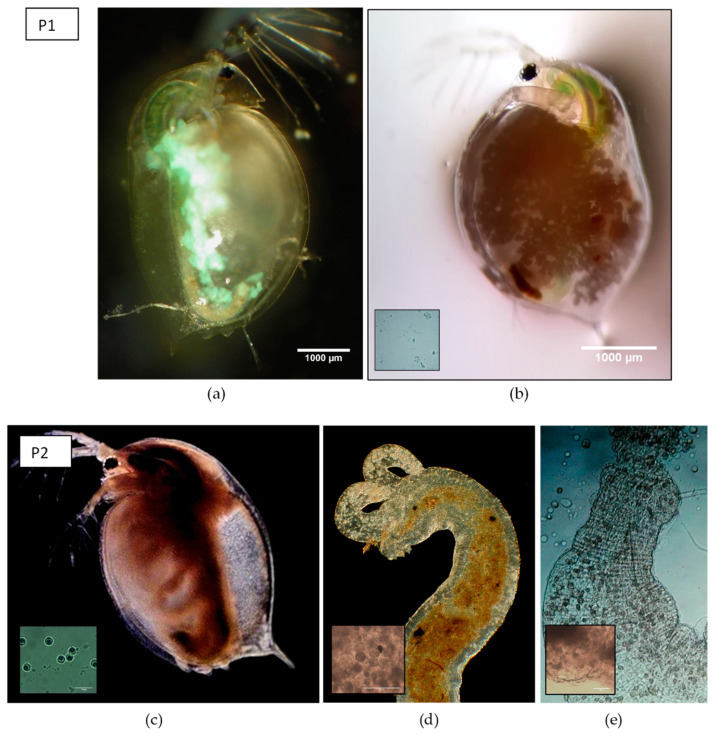
Overview of parasite species in the different parasite communities. (**a**) *Daphnia magna* individual infected with the causative agent of WFCD: Infected fat cells display a greenish, iridescent shine in reflected light, (**b**) heavily infected individual with *Binucleata daphniae* showing accumulated spores in the hemocoele of the carapace with detail of spore cluster, (**c**) individual infected with *Pasteuria ramosa* 30 days after exposure displaying a reddish appearance and larger body size (adapted photograph by Nina Schlotz, distributed under a Creative Commons Attribution 4.0 International license [51]), with detail of the final spore stages (400× magnification), (**d**) infected foregut with *Ordospora colligata*, (photograph by Dieter Ebert, distributed under a Creative Commons Attribution-Share Alike 4.0 International license) with detail of spore cluster (400× magnification), (**e**) infected hindgut with *Mitosporidium daphniae* with detail of spore cluster (100× magnification). Parasite treatment P1 consisted of a mixture of a and b, whereas parasite treatment P2 was composed of c, d, and e.

We first performed a microbiome adaptation experiment in which we pre-exposed *Daphnia* and their microbiomes to three types of parasite treatments. By doing so, we obtain gut microbiomes which communities have been altered by exposure to a parasite community. The gut microbiomes are then to be inoculated in fixed recipient genotypes (see further below). The experimental design consisted of *Daphnia* populations crossed with three parasite treatments (Figure 1), with three replicates per multifactorial combination. The parasite treatments consisted out of two different parasite communities and one control treatment. Parasite community 1 (further referred to as P1) consisted of a pool of the iridovirus causing White Fat Cell Disease (further referred to as WFCD, Figure 2a, [44]) and *Binucleata daphniae* (Figure 2b, [45]). WFCD is an iridovirus which infects the adipose tissue of *Daphnia* and is a highly virulent parasite, as it induces mortality in its host. Infection by WFCD is visible as a greenish, iridescent shine from the fat cells in reflected light. *Binucleata daphniae* is a microsporidian parasite known to infect the integument cells lining the hemocoel of its *D. magna* host. Infection with *B. daphniae* results in a reduced reproduction and survival. Parasite community 2 (further referred to as P2) consisted of a pool of *Pasteuria ramosa* (Figure 2c, [46,47]), *Ordospora colligata* (Figure 2d, [48]), and *Mitosporidium daphniae* (Figure 2e, [49]). *P. ramosa* is an obligate endospore-forming bacterial parasite in *Daphnia*, and it infects the hemocoel. Infection with *P. ramosa* often results in castration of the host (partial or complete stop of reproduction), indirectly resulting in an increase in *Daphnia* body size (i.e., gigantism). Infection with *P. ramosa* is also known to be highly genotype-specific and has little to no impact on survival. *O. colligata* and *M. daphniae* are both microsporidian parasites infecting the *D. magna* gut cells, with *O. colligata* infecting the foregut and *M. daphniae* the hindgut. Both endo-parasites are avirulent, as they induce small reductions in survival and reproduction success. Prevalence of these avirulent endoparasites can reach up to 100% in natural populations. All parasites used in this experiment (P1 and P2) are known to transmit horizontally, i.e., infection is not passed down from mother to offspring, but originates from the environment. The P1 pool was sampled from infected *D. magna* individuals from Blauwe hoeve in Kortrijk (50°48′57.8″ N 3°16′19.6″ E; WFCD and *B. daphniae*) and Muinkpark in Gent (51°02′33.1″ N 3°43′54.4″ E; WFCD). The P2 pool was sampled from infected *D. magna* individuals from Pottelberg pond in Aalbeke (50°47′01.6″ N 3°14′13.7″ E). The control treatment (PC) was not exposed to any parasite community. *Daphnia* populations receiving P1 and P2 originated from two natural haphazardly chosen ponds (the Kennedy Pond in Kortrijk, 50°48′05.9″ N 3°16′33.3″ E and the Morinne Pond in Kortrijk, 50°48′20.8″ N 3°18′45.2″ E). *Daphnia* populations receiving PC were pooled lab genotypes to avoid possible parasite influx from natural populations. All populations, the natural populations as well as the non-infected cultures in the laboratory, were exposed to a mixture of pond water from two *Daphnia*-free ponds to attempt a similar bacterioplankton community for all populations. Pond water was obtained from a mixture from the Kulak Pond (50°48′30.2″ N 3°17′38.3″ E) and the Libel Pond (50°47′44.3″ N 3°15′22.8″ E), both located in Kortrijk, Belgium. Pond water was subsequently filtered over 140 µm and 10 µm and pooled before exposing to the *Daphnia* populations from the microbiome adaptation experiment. Guts from the infected individuals in the microbiome adaptation experiment were dissected after infection reached its peak (after two weeks). This time period was based on research by Macke et al. [39] and Houwenhuyse et al. [50], which indicates a stable microbial gut community after perturbation by the biotic antagonist *Microcystis aeruginosa* after 7 days. To additionally ensure a stable community, we prolonged this period to the timepoint (2 weeks in this experiment) where infection rates started to decrease after reaching its peak. Dissected guts were utilized as microbial donor inocula for recipient *Daphnia* individuals in the microbiome transplant experiment. Microbial inocula were pooled per parasite treatment and per replicate vial. Each microbial pool inoculum was filtered over a sterile glass microfiber filter (grade GF/C; mesh size 1.1 µm) utilizing vacuum filtration to remove parasite spores from the microbial community. 

### 2.2. Microbiome Transplant Experiment

After obtaining the microbiome inocula, we performed the microbiome transplant experiment to examine the role of the gut microbiome on host tolerance upon parasite re-exposure. The experimental design consisted of three microbiome treatments × three parasite treatments × three recipient genotypes (Figure 2). Each multifactorial combination of microbiome treatment, parasite treatment, and genotype was replicated independently three times (*Daphnia* individuals were isolated from independently cultured maternal lines). Ultimately, six individuals per replicate were set up, which totals 486 *Daphnia* individuals. All individuals were made germ-free (0 days old; axenity performed via an adapted protocol of Callens et al. [8]; see Appendix A) and were individually placed in 20 mL sterile filtered tap water in closed off sterile vials. Each germ-free *Daphnia* individual (i.e., recipient) was inoculated with one of the microbial inocula (i.e., microbiome treatment: M1, M2, or MC), receiving the equivalent of 0.75 gut per *Daphnia* individual. As the experimental individuals displayed some mortality, we decided to further boost survival of all individuals. At day 3, all individuals were given a broader microbiome pool derived from non-infected whole-organism *Daphnia* individuals with the equivalent of 1 squashed *Daphnia* per 6 *Daphnia* individuals. By doing so, we expected to increase general performance of our *Daphnia* as prior administration of bacterioplankton with a highly diverse community to our stock *Daphnia* in the lab resulted in an increase in survival and reproduction (personal observation). Adding additional microbial strains to our vials could possibly interfere with our experimentally manipulated donor microbiome inocula. We do, however, assume little impact by adding these additional microbial communities as the community present in the donor microbial inocula already colonized the gut. Additionally, the boosting communities were pooled and given in equal quantities to all experimental individuals. In this manner, limited effects on the host microbiome are equal across all treatments. Additionally, all recipient individuals received the same amount and same composition of pooled microbiota. 

At day 5 and 6, all individuals were exposed to their respective parasite treatment (i.e., re-exposure with P1, P2, or PC). 5.8 × 10^3^ spores of *B. daphniae* for P1 and 1.4 × 10^3^ spores of *P. ramosa* for P2 were added per vial on the two consequent days. Spore solutions were obtained by squashing infected *Daphnia*. Spore counts for WFCD were not possible, as it is caused by an iridovirus [44], which cannot be routinely quantified under the microscope. Spore count for P2 was based on *P. ramosa* concentrations as this ensured sufficient exposure for infection, as spore counts for *O. colligata* and *M. daphniae* are generally higher compared to *P. ramosa* [52].

Prior to the exposure in the microbial transplant experiment, each parasitic inoculum was filtered over a sterile glass microfiber filter (grade GF/C according to the protocol as described for the microbiome filtration) to remove dominant contaminating microbiota from the parasite suspensions. In this manner, we want to examine the effect of the parasite community without possible interfering effects of the associated microbial strains. Measurement of the P1 suspension through qPCR showed that the microbial load in the parasite treatments was reduced to 19% due to the filtration. Although our parasite spore suspension were not axenic, we expect little impact on the recipient gut community as administered volume is low and prior to colonization from our microbiome inocula. Parasite spores were brought in resuspension after filtration by placing the filter in 15 mL of sterile distilled water and gently shaking the vial to optimize detachment of the spores from the filter. The obtained spore-solution was then administered to the respective recipient *Daphnia* individuals. For PC, a similar volume as the parasite treatments (P1 and P2) of filtered squashed non-infected individuals was added per vial. The parasite inocula utilized in this experiment were derived from the same parasite communities utilized in the microbiome adaptation experiment to infect *Daphnia*. In this manner, we compared the same parasite communities in the microbiome adaptation and microbiome transplant experiment.

*Daphnia* were given a relatively low daily amount of 0.5 mg C/L of axenic *Chlorella vulgaris* between day 0 to day 6 to ensure high uptake of microbiota and parasite spores for the microbiome exposure and parasite exposure, respectively. From day 7 onwards, *Daphnia* individuals received 1 mg C/L of axenic *C. vulgaris*. *C. vulgaris* (strain SAG 211-11 B) cultures were started from an axenic slent and cultured in sterile WC medium enriched with NaNO_3_ (425.05 mg/L) and K_2_HPO_4_.3H_2_O (43.55 mg/L) (adapted from [53]). The algae was cultured under sterile conditions in a climate chamber at 22 °C (±2 °C) and under fluorescent light (120 μmol·m^−2^·s^−1^) at a 16:8 h light:dark cycle in 2 L glass bottles. Algae cultures were maintained in batch cultures and were constantly stirred and aerated. Filters (0.22 µm) were placed at the input and output of the aeration system to avoid contamination. Algae were weekly harvested in stationary phase and checked for axenity via DAPI staining and LB and R2A medium agar plates.

The amount of sterile filtered tap water in each vial was increased from day three onwards on a daily base until 45 mL was reached. *Daphnia* individuals were monitored for survival and reproduction on a daily base. On day 11, all individuals were measured for their body size and their guts were dissected to analyse the microbial communities. Body size was defined as the distance between the head and the base of the tail. Visual screening for parasites or spores is generally possible two weeks after infection and not incorporated in this experiment. We, nonetheless, opted to dissect at day 11 to obtain sufficient gut microbial material as individuals were dying, and otherwise too little genetic material would be available for amplicon sequencing.

### 2.3. Statistical Analysis of Body Size and Survival

To examine *Daphnia* performance, we analyzed differences in body size. Body size was squared transformed. Normality of body size was tested for using a Shapiro–Wilk and Bartlett test. Differences in body size were analyzed using a nested linear mixed-effects model (LMER) with microbiome treatment, parasite treatment, and genotype as a fixed effect and maternal line as a random effect (Satterthwaite’s method). A Tukey HSD test was used to make post hoc pairwise comparisons. All statistical tests on body size were performed in R 4.0.2 [54]. Analyses on reproduction were not possible due to few data points, as the experiment ended at the age of maturation. 

To examine *Daphnia* tolerance and performance, we analyzed differences in survival. Survival was analyzed with a Cox proportional-hazards model regression using the SAS 9.4 software (PHREG procedure). Genotype, microbiome treatment, and parasite treatment were specified as fixed factors. The survival times of individuals that were still alive at the end of the experiment were coded as censored. As ties in survival were numerous, the Efron approximate likelihood was applied. Pairwise comparisons were performed using the CONTRAST statement, which provided both the hazard ratios (HR) between groups for the variable of interest, and the associated p-values. Survival curves were obtained with the ggsurvplot() function [Survminer package] in R 4.0.2 [54].

### 2.4. MiSeq Library Preparation

To identify the bacterial composition present in the gut, the guts of the surviving *Daphnia* per replicate were dissected under a stereo-microscope with sterile dissecting needles at the end of the experiment and pooled per replicate (mean = 4.309 guts/sample; sd = 1.357 guts/sample; Table S1). Pearson correlations were executed between the number of sequenced guts and the alpha diversity-diversity variables to check for interdependence. Genotype, microbiome treatment, parasite treatment, all two-way interactions, and the three-way interaction, all showed no significant correlation, dismissing the issue of interdependence (Table S2).

Guts were transferred to 10 µl of sterile MilliQ water. Samples were stored under −20 °C until further processing. DNA was extracted using a PowerSoil DNA isolation kit (MO BIO laboratories, Carlsbad, CA, USA) and dissolved in 20 μL MilliQ water. The total DNA yield was determined using a Qubit dsDNA HS assay (Invitrogen, Merelbeke, Belgium) on 1 μL of sample. Because of initially low bacterial DNA concentrations, a nested PCR was applied to increase specificity and amplicon. For the external amplification, a PCR reaction was run using primers 27F and 1492R on all of the template (98 °C-30 s; 98 °C-10 s, 50 °C-45 s, and 72 °C-30 s; 30 cycles; 72 °C-5 min; 4 °C-hold) using the Platinum SuperFi DNA polymerase (Thermofisher, Merelbeke, Belgium). PCR products were subsequently purified using the QIAquick PCR purification kit (Qiagen, Antwerp, Belgium). To obtain dual-index amplicons of the V4 region, an internal PCR was performed on 5 µL of PCR product using a unique combination of a forward and revers primer per sample (Table S3; 98 °C-30 s; 98 °C-10 s, 55 °C-45 s and 72 °C-30 s; 30 cycles; 72 °C-5 min; 4 °C-hold). Both primers contained an Illumina adapter and an 8-nucleotide (nt) barcode at the 5′-end. For each sample, PCRs were performed in triplicate, pooled, and gel-purified using the QIAquick gel extraction kit (Qiagen). An equimolar library was prepared by normalizing amplicon concentrations with a SequalPrep Normalization Plate (Applied Biosystems, Geel, Belgium) and subsequent pooling to standardize DNA concentrations. Amplicons were sequenced using a v2 PE500 kit with custom primers on the Illumina Miseq platform (KU Leuven Genomics Core, Leuven, Belgium), producing 2 × 250-nt paired-end reads.

### 2.5. Analysis of Microbial Communities

Sequence reads, statistical analyses, and plots were performed using R 4.0.2 [54] following [55]. Sequences were trimmed (the first 10 nucleotides and all nucleotides from position 190 onward were removed) and filtered (maximum two expected errors per read) on paired ends jointly. Sequence variants were inferred using the high-resolution DADA2 method [56,57], and chimeras were removed. Taxonomy was assigned with a naive Bayesian classifier using the Silva v132 training set. Amplicon sequence variants (ASVs, hereafter called OTUs) which had no taxonomic assignment at phylum level or were assigned as “Chloroplast” or “Cyanobacteria” were removed from the dataset. The final recipient dataset contained, after trimming, 2,295,594 reads with an average of 31,883,25 reads per sample (minimum = 1512 reads, maximum = 102,065 reads).

To examine differences in community composition within the different recipient samples and variables, alpha diversity was determined by calculating OTU richness and Shannon diversity number using the vegan package in R [58] following [59]. OTU richness was calculated as the sum of the present OTUs. Shannon diversity number was calculated as the exponential function of Shannon entropy and will be further referred to in this text as ‘Shannon entropy’. First, all samples were rarified to a depth of 10,000 reads. The effect of genotype, microbiome treatment, and parasite treatment on OTU richness and Shannon entropy’ was examined using a generalized linear model assuming a Poisson distribution of the data and accounted for overdispersion, as the observed residual deviance was higher than the degrees of freedom. Pairwise comparisons among significant variables and their interactions were performed by contrasting least-squares means with Tukey adjustment. To examine differences in gut microbial community composition among samples, a Bray–Curtis dissimilarity matrix was calculated. Differences between main effects and interaction were examined through a permutation MANOVA (Adonis function, vegan package). Multivariate community responses to genotype and treatments were investigated by means of Principal Coordinates Analysis. Obtained *p*-values were adjusted for multiple comparisons through the control of the false discovery rate (FDR). To identify which bacterial classes differed significantly between main and interaction effects, differential abundance analyses were performed (DESeq2 function) on the raw sequencing data from which low data counts were removed (less than 1000). Additionally, donor samples were analyzed to compare alpha diversity and overlap between donor and recipients and within the donors. Due to loss of samples, only three samples from the donor P1 treatment and one sample from the donor P2 treatment were recovered.

## 3. Results

### 3.1. Effect on Recipient Daphnia Tolerance and Performance in Terms of Body Size and Survival

**Figure 3:**
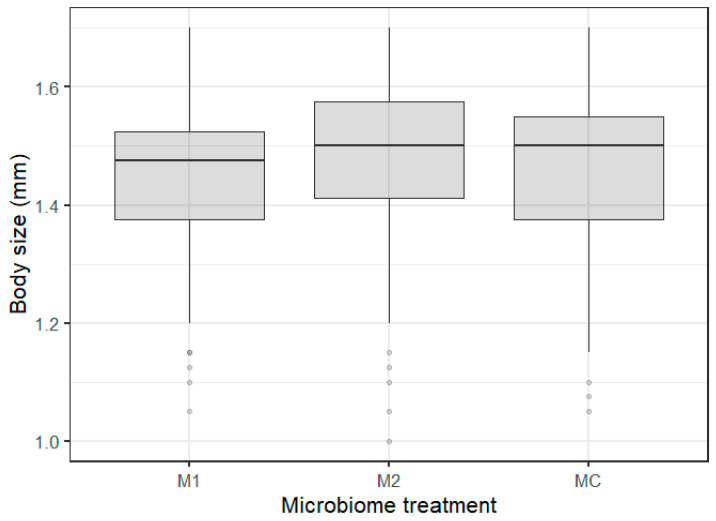
Boxplots of *Daphnia* body size exposed to the different microbiome treatments (M1, M2, and MC).

The differences in body size were best explained by the microbiome inocula treatment (Table 1). Within the microbiome inocula treatments, *Daphnia* exposed to microbiome treatment 1 (M1, pre-exposure to WFCD + *B. daphniae*) were significantly smaller (Figure 3; Table S4) than animals exposed to microbiome treatment 2 (M2, pre-exposure to *P. ramosa* + *O. colligata* + *M. daphniae*). No significant differences between M1 and MC, and M2 and MC treatments were observed (Table S4, Figure 3). There was no role of the microbiome upon parasite re-exposure as no significant microbiome x parasite interaction was observed for body size and survival (Table 1).

**Figure 4:**
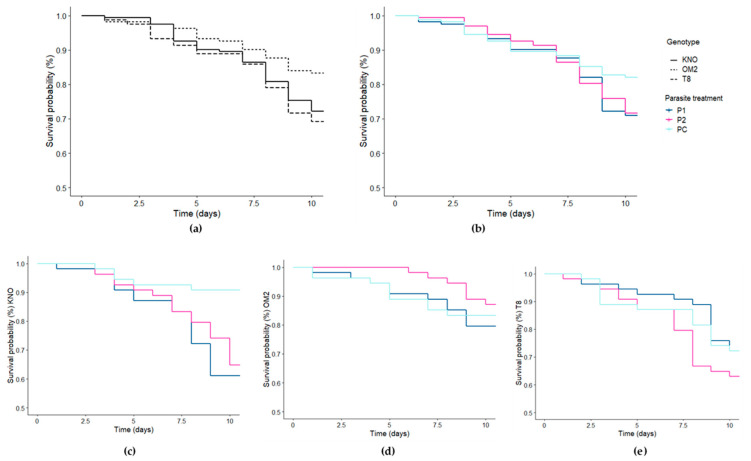
Survival curves for the *Daphnia* individuals for the different (**a**) genotypes, (**b**) parasite community treatment, (**c**) parasite community treatment for the KNO genotype, (**d**) parasite community treatment for the OM2 genotype, and (**e**) parasite community treatment for the T8 genotype. Line type indicates the genotype (solid = KNO, dotted = OM2, dashed = T8). Line color indicates the parasite treatment (dark blue = P1, pink = P2, light blue = PC).

The differences in *Daphnia* survival were best explained by the genotype, parasite community treatment, and the *Daphnia* genotype × parasite community treatment interaction (Table 1; Table S4). Within the KNO genotype, *Daphnia* receiving the P1 or P2 treatment survived significantly shorter than *Daphnia* receiving the PC treatment (Figure 4; Table S4). Within the OM2 and T8 genotypes, none of the parasite treatments induced a significant reduction in *Daphnia* survival (Figure 4; Table S4). Within the P1 and P2 treatment, OM2 individuals survived significantly longer than the KNO and T8 individuals (Figure 4; Table S4). Within the PC treatment, KNO individuals survived significantly longer than T8 individuals (Figure 4; Table S4). Within the main genotype effect, OM2 individuals had a significantly higher survival compared with both T8 and KNO individuals (Table S4; Figure 4). Within the main parasite effect, individuals receiving the control treatment had a significantly higher survival compared with individuals receiving a parasite treatment (Table S4). There was no significant microbiome x parasite interaction (Table 1).

### 3.2. Characterisation and Experimental Treatment Effects on Recipient Daphnia Gut Microbial Communities

**Figure 5:**
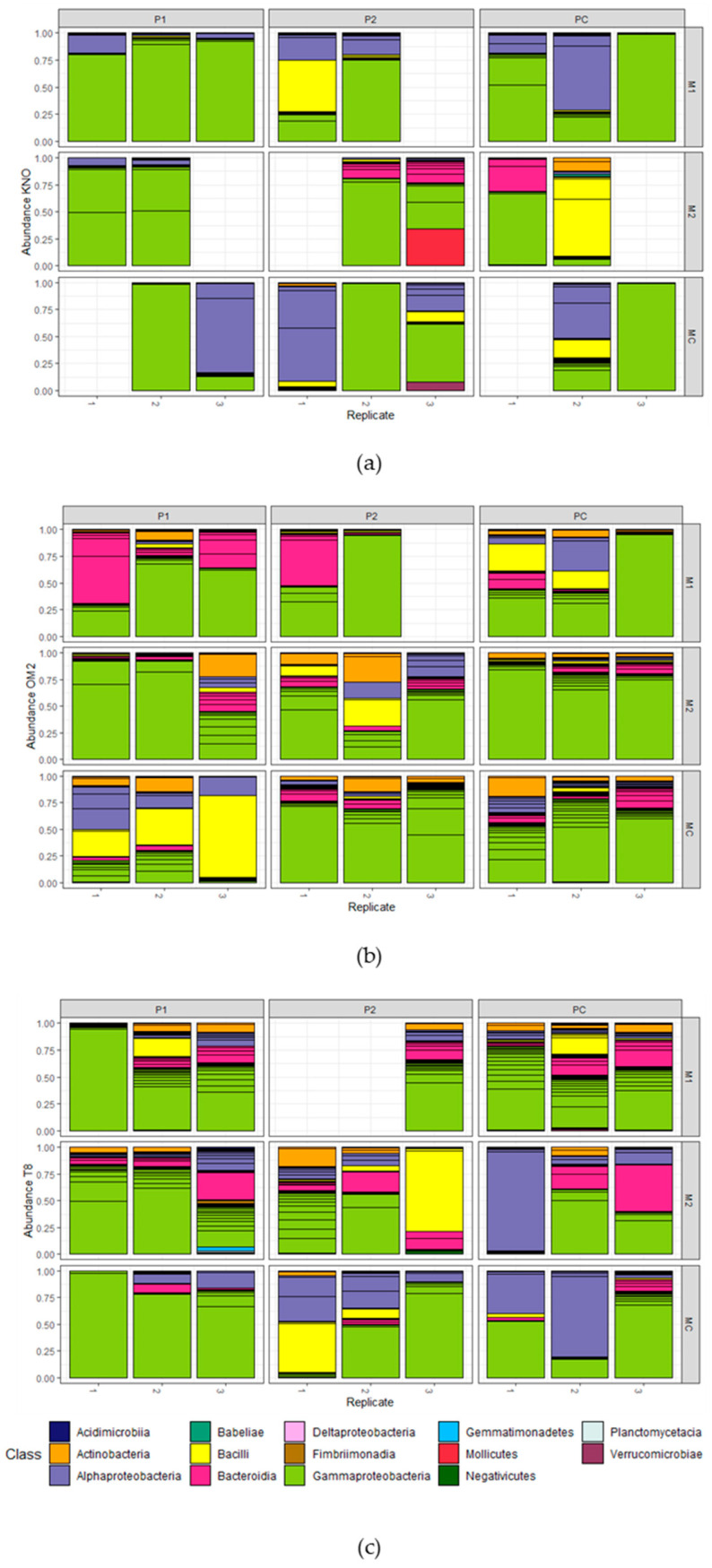
Gut microbial composition of *Daphnia* in the microbiome transplant experiment. OTU relative abundances are portrayed for (**a**) the KNO genotype, (**b**) the OM2 genotype, and (**c**) the T8 genotype. Recipient populations are grouped per multifactorial combination of microbiome and parasite community. Colors indicate the bacterial class. OTUs with a relative abundance lower than 1% are not included. Each bar represents one sample containing gut material of one to six recipients which was pooled for analysis.

Composition of the gut microbiomes: Eleven days after the microbial inoculation in the recipient *Daphnia*, the gut microbiomes were mainly dominated by Gammaproteobacteria (mean = 61.91%; sd = 29.00%), followed by Alphaproteobacteria (mean = 15.13%; sd = 21.87%), Bacteroidia (mean = 9.22%; sd = 12.55%), and Bacilli (mean = 8.40%; sd = 17.50%; Figure 5). The most dominant OTUs in all samples were Burkholderiaceae sp. (mean = 29.94%; sd = 28.01%; Gammaproteobacteria), *Methylobacterium* sp. (mean = 6.59%; sd = 15.64%; alphaproteobacteria), and *Streptococcus* sp. (mean = 6.09%; sd = 13.60%; Bacilli; Table S5). Gammaproteobacteria, more in particular, the Burkholderiaceae sp., were the most abundant taxa across all main treatments and two-way interactions. Interestingly, samples derived from M2 exposed individuals contained all sequenced classes, whereas all other treatments lacked one or more classes. Samples derived from OM2 individuals and P1 exposed individuals contained all sequenced classes, except Babeliae, Fimbriimonadia, and Gemmatimonadetes. Results of the Log2Fold test on the main effects on OTU level can be found in Table S6.

#### 3.2.1. Alpha Diversity of Recipient Daphnia Gut Microbiomes

**Figure 6:**
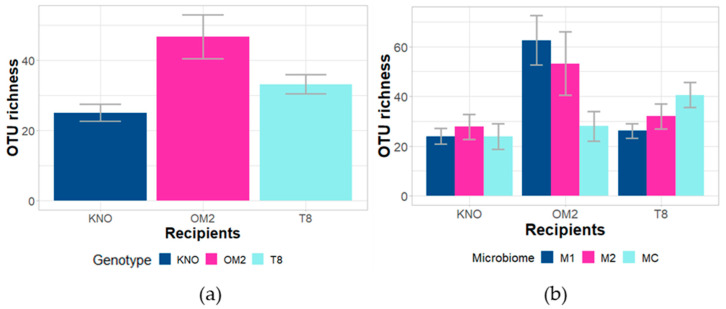
Effect of (**a**) genotype and (**b**) genotype × microbiome interaction for recipient samples on OTU richness. Error bars indicate standard error.

The differences in recipient OTU richness were best explained by *Daphnia* genotype and the *Daphnia* genotype × microbiome inoculum interaction (Figure 6; Table 1). Within the OM2 genotype, the microbial alpha diversity in the M1 treatment was significantly higher than the MC treatment (Table S4; Figure 6) before applying FDR correction. After applying FDR correction, no significant differences within all three genotypes were observed (Table S4). When examining the main genotype effect, OTU richness of the recipient guts of the KNO genotype was significantly lower compared with the OM2 genotype. No significant differences within the T8 genotype were observed: T8 showed a non-significant intermediate OTU richness. The differences in Shannon entropy’ were best explained by *Daphnia* genotype, *Daphnia* genotype × microbiome inoculum interaction, and microbiome inoculum × parasite community interaction (Table 1). Within the OM2 genotype, the M1 treatment was significantly higher compared than the MC treatment before correction (Table S4). After applying FDR correction, no significant differences within all three genotypes were observed (Table S4). No significant differences were observed within the microbiome inoculum × parasite community interaction for the Shannon entropy’. When examining the main genotype effect, the Shannon entropy’ for the KNO genotype was significantly lower compared with the OM2 genotype (Table S4). Results of analyses including the donor samples can be found in Figure S2.

#### 3.2.2. Treatment Effects on Gut Microbial Community Composition

**Figure 7:**
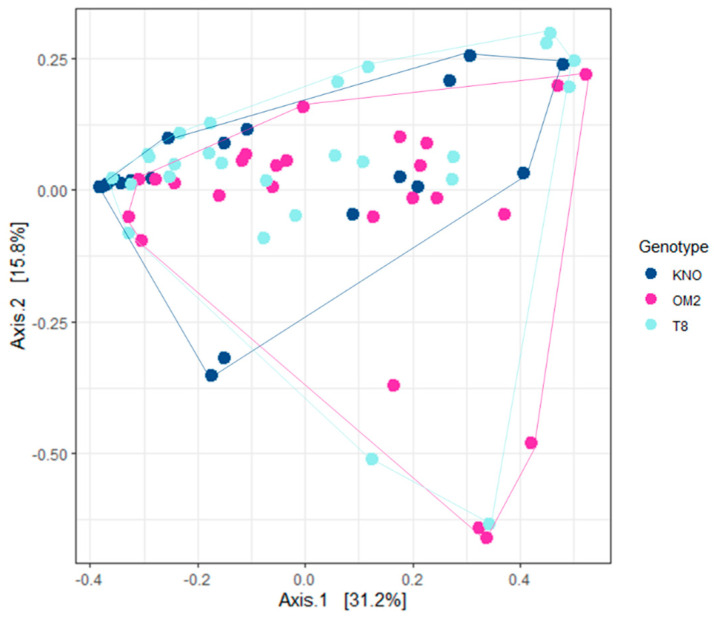
PCA of the gut microbial communities of recipients using weighted UniFrac distance for genotype. Colors indicate genotype.

Variation in gut microbial community composition was mainly explained by *Daphnia* genotype (Figure 7; Table 1). KNO differed significantly from OM2 and T8 (Table S4). Bray–Curtis ordinations demonstrated a complete overlap between OM2 and T8 individuals, indicating that the bacterial community of the OM2 and T8 genotype was similarly structured. KNO, on the other hand, showed complete overlap with OM2 and T8 individuals, but grouped closer together, suggesting a more homogeneous community between the different individuals in KNO than in OM2 and T8. Results of analyses including the donor samples can be found in Figure S3.

## 4. Discussion

We here investigated the role of the gut microbiome on host tolerance upon parasite re-exposure through a microbiome adaptation and microbiome transplant experiment in germ-free individuals. We compared a pool of an iridovirus and a microsporidian parasite, and a pool of an endobacterium (*P. ramosa*, cfr. [38]) and two microsporidian parasites with a control. These gut microbiomes were obtained and used as microbial inocula (donors) in the microbiome transplant experiment. Germ-free *Daphnia* in our microbiome transplant experiment (recipients) received the gut microbiome inocula and were then exposed to the same three parasite treatments. Additionally, we examined intraspecific responses by including three *Daphnia* genotypes in our study. We here, thus, focus on a broader pool of parasites and on parasite-induced virulence effects than in Sison-Mangus et al. [38], who focused on parasite resistance for *P. ramosa*. Additionally, we included parasite re-exposure, whereas Sison-Mangus et al. [38] focused on initial parasite exposure. In conclusion, we aimed to examine the role of the gut microbiome on host tolerance upon parasite re-exposure in our recipients. We expected that the gut microbiome would affect *Daphnia* performance (survival and body size) in our recipients upon re-exposure to these parasite communities and that parasite specific responses could be detected. Three outcomes were thus possible upon re-exposure to the same parasite community: (1) No role of the microbiome, (2) a negative role of the microbiome, reflected in a reduced tolerance in case of parasite-mediated dysbiosis through the microbiome, or (3) a positive role of the microbiome, reflected in an improved tolerance of the gut microbiomes in case certain beneficial bacterial strains were selected for. Our results showed that (i) the gut microbiome plays no role in mediating *Daphnia* tolerance towards certain parasite communities upon re-exposure, (ii) the gut microbiome community affected *Daphnia* body size in a parasite specific way, (iii) it is the genotype rather than the microbiome affecting *Daphnia* survival, and (iv) *Daphnia* genotype plays an important role in shaping the gut microbiome community, both in alpha diversity and in the composition.

No microbiome × parasite effect was found on *Daphnia* body size, which implies that the gut microbiome has no substantial impact on body size upon parasite re-exposure. We did find that variation in *Daphnia* body size was dependent on the main gut microbiome inoculum treatment reflecting parasite specific responses (of the donors). Individuals exposed to gut microbiomes extracted from individuals exposed to the WFCD-*B. daphniae* pool (M1) were significantly smaller compared with individuals exposed to the gut microbiomes extracted from individuals exposed to the *P. ramosa-O. colligata-M. daphniae* pool (M2). This was surprising at first, as we assumed body size to be different in the recipients receiving the parasite pre-exposed gut microbiomes compared with the individuals receiving the control gut microbiomes, which would reflect parasite induced dysbiosis. This difference in body size between M1- and M2-exposed individuals could possibly be due to the presence of particular microbial communities (more or less diverse) or of particular bacterial strains associated with these parasite communities. Interestingly, *P. ramosa* (present in M2) is known to induce gigantism, i.e., an increased body size in infected *Daphnia* [37,60]. Alternatively, the presence of the less virulent (sometimes even mutualistic) microsporidian gut parasites may have induced protection in the P2 and M2 treatments, an effect which has been suggested in [61], especially in low food quality conditions. The amplicon sequencing results should reveal possible links between *Daphnia* body size and gut microbial communities. Studies have shown that an increased diverse bacterial community in the gut can be associated with a larger body size in *Daphnia*, e.g., [50,62]. Similarly, reduced growth and metabolic capacities due to parasite-induced reduced gut bacterial diversity has been observed in other species, such as mice [63]. The amplicon sequencing analysis reflected a lower alpha diversity in the inocula, but did not show a significantly reduced alpha diversity in the M1 treatment compared with the M2 and MC treatment in the recipients. Our amplicon analysis did reveal that M1 exposed individuals had a significantly lower proportion of, e.g., *Methylobacterium* sp. (Gammaproteobacteria) in their gut compared with M2 and MC exposed individuals. These body size related results imply that the gut microbiome plays an additional role in the already complex food web *Daphnia* is a part of in freshwater populations, especially because multiple infections often occur [52]. Body size is a critical trait in shaping consumer–prey interactions. A priori parasite exposure will alter the gut microbial community, impacting *Daphnia* body size, and as such impact, e.g., grazing and predation by zooplanktivorous fish.

No microbiome × parasite effect was found on *Daphnia* survival, which implies the gut microbiome has no substantial impact on survival size upon parasite re-exposure. Additionally, no main microbiome effect was detected. Our results on *Daphnia* performance thus suggest that the microbiome plays no role on *Daphnia* tolerance upon parasite re-exposure. This is in contrast with studies on, e.g., bumblebees which show a reciprocal role between the gut microbiome and parasite exposure [9,11,31]. The study by Sison-Mangus et al. [38], however, also found no evidence on a gut microbial-mediated resistance against the parasite *P. ramosa*. It appears that host regulated defense against parasites in *D. magna* is mediated in lesser extent or even completely independent from the gut microbial community. Interestingly, these results also suggest that exposure can alter gut microbial communities, but not in a selective manner resulting in, e.g., an improved tolerance towards certain parasites. Even though an increasing amount of studies hints at a protective role of the gut microbiome when encountering parasite infection, our results attenuate its assumed protective role. In our study, host tolerance is dependent on host genotype [64], independent of the gut microbial community. We were also interested in intraspecific differences, which is why we incorporated three genotypes in this study. We found a strong *Daphnia* genotype effect on survival as the genotype × parasite community interaction, as well as the main genotype effect significantly impacted *Daphnia* survival. These results are in line with previous studies which report strong genotype × genotype host–parasite [47,65,66] or *Daphnia* genotype × parasite species interactions [64]. It appears that, in our study, the KNO genotype thrives best under control conditions, whereas the OM2 genotype suffered the least from the exploitation by the parasite communities compared with the KNO and T8 genotypes.

Host genotype does not only mediate host tolerance in terms of survival, but also appears to be a strong determinant of the *Daphnia* recipient gut microbiome. Firstly, we found a significant effect on alpha diversity (OTU richness in our study), both from the genotype × microbiome interaction as the main genotype effect. Secondly, we found a significant main genotype effect on the microbial community composition of the *Daphnia* gut. These effects can be attributed to different immune system pathway expressions of the *Daphnia* genotypes, e.g., innate immune system genes expression or the production of antimicrobial peptides structuring the gut microbiome [67], or due to differences in molting capacities affecting bacterial establishment [68], amongst others. The importance of the host genotype in shaping the gut microbial composition has also been suggested by correlational studies on humans and mice in which correlations between the gut microbiome and genes associated with diet, innate immunity, vitamin D receptors, and metabolism were revealed [69,70]. Host genotype can also reciprocally influence the microbial community composition. Genotype-specific gut microbiomes can also be found in, e.g., sponges [71], corals [72], and mice [73]. These studies also revealed a stronger role of host genotype compared with the environment [72] and sex [73] in driving gut microbial variation. Further investigations are necessary to get insights into the mechanisms behind these genotype-specific gut microbiomes. Interestingly with this respect is that *Daphnia* genotypes can display different grazing behavior, resulting in different feeding patterns and consumed bacteria [42,43,74,75]. Our results suggest that the *Daphnia* genotype is a stronger determinant of gut microbial alpha diversity than the pre-exposure of the microbial inocula. The main genotype effect reveals that the gut microbial composition was structured by the presence of particular OTUs. OM2 individuals differed significantly from both the KNO and T8 individuals in relative abundance of the OTU *Methylobacterium* sp., which has the highest abundance in the OM2 genotype. This increased proportion of the *Methylobacterium* genus is also observed in M2 individuals, which had a higher body size compared with M1 individuals. Mono-association experiments with *Methylobacterium* sp. could give us more insights in its role of this OTU in *Daphnia* functioning. The amplicon sequencing analyses revealed an effect of the genotype on the gut microbial communities using Bray–Curtis dissimilarity and weighted UniFrac distance.

## 5. Conclusions

In conclusion, we can state that the microbiome plays no role in mediating *Daphnia* tolerance upon parasite re-exposure in our study. Our study does suggest an impact of the gut microbial community on body size, reflecting parasite specific responses. We found that it was rather the *Daphnia* genotype which mediated *Daphnia* tolerance, as survival upon re-exposure was mainly determined by the host genotype. Additionally, our study suggests a host genotype-specific gut bacterial community on alpha diversity, microbial community composition, and on the presence of specific strains.

## Figures and Tables

**Table 1:** Significant results of the statistical analysis on the effect of genotype, microbiome treatment, parasite community treatment, and their interactions on body size, survival, and alpha diversity variables (OTU richness, Shannon entropy’). Degree of freedom (DF) is indicated per main and interaction effect. Obtained *p*-values were adjusted for multiple comparisons through the control of the false discovery rate (FDR).

		Adjusted *p*-Value				
	DF	Body Size	Survival	OTU Richness	Shannon Entropy’	Microbial Community Composition
Genotype	2		<0.001	<0.001	<0.001	0.014
Microbiome treatment	2	0.034				
Parasite treatment	2		0.002			
Genotype x Microbiome treatment	4			0.015	0.004	
Genotype x Parasite treatment	4		0.003			
Microbiome treatment x Parasite treatment	4				0.047	
Genotype x Microbiome treatment x Parasite treatment	8					

## Data Availability

The datasets generated for this study can be found in the NCBI, under accession number PRJNA688519.

## Supplementary Materials

The following are available online at https://www.mdpi.com/2073-4425/12/1/70/s1, Table S1: Overview of the number of pooled recipient guts per microbial sample, Table S2: Overview results for Pearson correlation between the number of guts per sample and OTU richness or Shannon entropy’. Raw and adjusted (adjusted for multiple comparisons through the control of the false discovery rate (FDR)) *p*-values are given, Table S3: Overview forward and reverse primers used for the internal PCR, Table S4: Results of post-hoc analyses on body size, survival, OTU richness, Shannon entropy’, and microbial community composition for the significant results of the statistical analysis (see Table 1). Raw and adjusted (adjusted for multiple comparisons through the control of the false discovery rate (FDR)) *p*-values are given for the results on *Daphnia* gut microbial communities, Table S5: Overview of relative abundances of the 40 most common OTUs in *Daphnia* guts from the microbiome transplant experiment. Abundances were calculated on rarefied data. Sd: Standard deviation, Table S6: Results Deseq analysis on class level and OTU level between main effects, Table S7: Significant results of the statistical analysis on the effect of genotype, microbiome treatment, parasite community treatment, and their interactions on body size, survival, and alpha-diversity variables (OTU richness, Shannon entropy’). Obtained P-values were adjusted for multiple comparisons through the control of the false discovery rate (FDR). Significant data (*p* < 0.05) is indicated in bold. Highly significant data (*p* < 0.001) are underlined. Raw and adjusted (adjusted for multiple comparisons through the control of the false discovery rate (FDR)) *p*-values are given for the results on *Daphnia* gut microbial communities. Figure S1: Effect of the genotype x parasite interaction on OTU richness. Colors indicate the different genotypes. Error bars indicate standard error. Figure S2: Effect of microbiome (M1 and M2) for donor and recipient samples on OTU richness. Error bars indicate standard error. Colors indicate the different microbiome treatments. OTU richness in the M1 inoculum (mean = 38.333, sd = 13.051) was, on average, lower compared with the M2 inoculum (OTU richness = 47.000). Figure S3: PCA of the gut microbial communities of recipients using weighted UniFrac distance for the donors (P1 and P2) and matching recipients (M1 x P1, M2 x P2) using weighted UniFrac distance for donor/recipient type and parasite treatment. Analyses on donor (P1 and P2) and matching recipient (M1 x P1, M2 x P2) bacterial communities showed a significant difference in structure for P1 (*p* = 0.005; R2 = 0.147), but not for P2 (*p* = 0.783; R2 = 0.330). Bray–Curtis ordinations revealed that both P1 and P2 showed complete segregation between donors and recipients (Figure 7b), indicating that the donors and recipients for both the P1 and P2 treatment were differently structured (Figure 7b), however non-significant for P2.

## Appendix A

### Appendix A.1. Daphnia Magna Genotypes

Three genotypes of *D. magna* were utilized in our transplant experiment: OM2 11.3; KNO 15.4; and T8 (further referred to as OM2, KNO and T8). The genotype OM2 was originally isolated from “Oude Meren, Abdij van‘t park” from Leuven in Belgium (50°51′47.82” N, 04°43′05.16” E). The genotype KNO was originally isolated from a small pond (350 m²) from Knokke in Belgium (51°20′05.62” N, 03°20′53.63” E). The genotype T8 was originally isolated from a shallow manmade pond from Oud Heverlee in Belgium (50°50′ N, 4°39′ E). All genotypes were maintained in the lab under standard stock conditions for several years prior to the transplant experiment. All genotypes were cultured in filtered tap water at a temperature of 19 °C (±1 °C) and under a 16:8 h light:dark cycle in 2L jars. They were fed every other day with a saturating amount of *Chlorella vulgaris*. Medium was refreshed every week. As all genotypes were hatched in the laboratory from resting eggs, and maintained in the laboratory for several years, it is unlikely that the experimental genotypes contain parasites and bacteria from the pond of origin.

### Appendix A.2. Preparation Maternal Lines

For each genotype, three maternal lines were set up. Five individuals per maternal line were transferred in 500 mL filtered tap water and fed every day with a saturating amount of *Chlorella vulgaris*. Medium was refreshed every week. Juveniles from the first brood were discarded. Twenty juveniles from the second brood per maternal line were transferred in 2l jars of filtered tap water. Maternal *Daphnia* were discarded after releasing their second brood. This process was repeated for the every new batch of 2nd brood juveniles up to seven generations.

### Appendix A.3. Preparation Axenic Daphnia

Recipient *Daphnia* were obtained from the maternal lines of OM2, KNO, and T8. Females carrying parthenogenetic eggs (second brood) were dissected under a stereomicroscope. Eggs which were no longer than 24 h deposited in the brood chamber were collected in sterile filtered tap water (n = 100 eggs per maternal line). These eggs were then disinfected under a laminar flow hood following an adjusted protocol of Callens et al. [8]. Eggs were placed in 6 mL of a 0.10% gluteraldehyde solution and gently agitated for 10 min. After this disinfection step, two washing steps were performed in which the eggs were transferred to sterile filtered tap water for each 10 min. Eggs were then transferred to six-well (cell culture) sterile plates, each well containing 6 mL of sterile filtered tap water, and incubated at 20 ± 0.5 °C. Eggs were allowed to hatch during 72 h under sterile conditions, and the resulting germ-free juveniles were used as recipients in the transplant experiment.
