# Supplementary material for: The Role of Microbiome and Genotype in Daphnia magna upon Parasite Re-Exposure
Source: Genes (Basel). 2021 Jan 7;12(1):70. doi: 10.3390/genes12010070 (PMC7825712; doi:10.3390/genes12010070)
Supplement: Supplementary file 1 [file genes-12-00070-s001.pdf]

Supplementary information

Table S1: Overview of the number of pooled recipient guts per microbial sample

Genotype	Microbiome	Parasite	KNOxM	CxP	MxP	CxMxP	Replicate	Id	Guts
KNO	M1	P1	KNO_M1	C_P1	M1_P1	C_M1_P1	1	C_M1_P1_1	4
KNO	M1	P1	KNO_M1	C_P1	M1_P1	C_M1_P1	2	C_M1_P1_2	6
KNO	M1	P1	KNO_M1	C_P1	M1_P1	C_M1_P1	3	C_M1_P1_3	1
KNO	M1	P2	KNO_M1	C_P2	M1_P2	C_M1_P2	1	C_M1_P2_1	4
KNO	M1	P2	KNO_M1	C_P2	M1_P2	C_M1_P2	2	C_M1_P2_2	3
KNO	M1	P2	KNO_M1	C_P2	M1_P2	C_M1_P2	3	C_M1_P2_3	0
KNO	M1	MC	KNO_M1	C_PC	M1_PC	C_M1_PC	1	C_M1_PC_1	6
KNO	M1	MC	KNO_M1	C_PC	M1_PC	C_M1_PC	2	C_M1_PC_2	4
KNO	M1	MC	KNO_M1	C_PC	M1_PC	C_M1_PC	3	C_M1_PC_3	5
KNO	M2	P1	KNO_M2	C_P1	M2_P1	C_M2_P1	1	C_M2_P1_1	3
KNO	M2	P1	KNO_M2	C_P1	M2_P1	C_M2_P1	2	C_M2_P1_2	4
KNO	M2	P1	KNO_M2	C_P1	M2_P1	C_M2_P1	3	C_M2_P1_3	0
KNO	M2	P2	KNO_M2	C_P2	M2_P2	C_M2_P2	1	C_M2_P2_1	2
KNO	M2	P2	KNO_M2	C_P2	M2_P2	C_M2_P2	2	C_M2_P2_2	6
KNO	M2	P2	KNO_M2	C_P2	M2_P2	C_M2_P2	3	C_M2_P2_3	3
KNO	M2	MC	KNO_M2	C_PC	M2_PC	C_M2_PC	1	C_M2_PC_1	6
KNO	M2	MC	KNO_M2	C_PC	M2_PC	C_M2_PC	2	C_M2_PC_2	6
KNO	M2	MC	KNO_M2	C_PC	M2_PC	C_M2_PC	3	C_M2_PC_3	6
KNO	MC	P1	KNO_MC	C_P1	MC_P1	C_MC_P1	1	C_MC_P1_1	3
KNO	MC	P1	KNO_MC	C_P1	MC_P1	C_MC_P1	2	C_MC_P1_2	6
KNO	MC	P1	KNO_MC	C_P1	MC_P1	C_MC_P1	3	C_MC_P1_3	3
KNO	MC	P2	KNO_MC	C_P2	MC_P2	C_MC_P2	1	C_MC_P2_1	4
KNO	MC	P2	KNO_MC	C_P2	MC_P2	C_MC_P2	2	C_MC_P2_2	5
KNO	MC	P2	KNO_MC	C_P2	MC_P2	C_MC_P2	3	C_MC_P2_3	3
KNO	MC	MC	KNO_MC	C_PC	MC_PC	C_MC_PC	1	C_MC_PC_1	5
KNO	MC	MC	KNO_MC	C_PC	MC_PC	C_MC_PC	2	C_MC_PC_2	5
KNO	MC	MC	KNO_MC	C_PC	MC_PC	C_MC_PC	3	C_MC_PC_3	5
OM2	M1	P1	OM2_M1	OM2_P1	M1_P1	OM2_M1_P1	1	OM2_M1_P1_1	4
OM2	M1	P1	OM2_M1	OM2_P1	M1_P1	OM2_M1_P1	2	OM2_M1_P1_2	4

OM2	M1	P1	OM2_M1	OM2_P1	M1_P1	OM2_M1_P1	3	OM2_M1_P1_3	6
OM2	M1	P2	OM2_M1	OM2_P2	M1_P2	OM2_M1_P2	1	OM2_M1_P2_1	5
OM2	M1	P2	OM2_M1	OM2_P2	M1_P2	OM2_M1_P2	2	OM2_M1_P2_2	5
OM2	M1	P2	OM2_M1	OM2_P2	M1_P2	OM2_M1_P2	3	OM2_M1_P2_3	5
OM2	M1	MC	OM2_M1	OM2_PC	M1_PC	OM2_M1_PC	1	OM2_M1_PC_1	5
OM2	M1	MC	OM2_M1	OM2_PC	M1_PC	OM2_M1_PC	2	OM2_M1_PC_2	4
OM2	M1	MC	OM2_M1	OM2_PC	M1_PC	OM2_M1_PC	3	OM2_M1_PC_3	5
OM2	M2	P1	OM2_M2	OM2_P1	M2_P1	OM2_M2_P1	1	OM2_M2_P1_1	4
OM2	M2	P1	OM2_M2	OM2_P1	M2_P1	OM2_M2_P1	2	OM2_M2_P1_2	5
OM2	M2	P1	OM2_M2	OM2_P1	M2_P1	OM2_M2_P1	3	OM2_M2_P1_3	5
OM2	M2	P2	OM2_M2	OM2_P2	M2_P2	OM2_M2_P2	1	OM2_M2_P2_1	5
OM2	M2	P2	OM2_M2	OM2_P2	M2_P2	OM2_M2_P2	2	OM2_M2_P2_2	6
OM2	M2	P2	OM2_M2	OM2_P2	M2_P2	OM2_M2_P2	3	OM2_M2_P2_3	5
OM2	M2	MC	OM2_M2	OM2_PC	M2_PC	OM2_M2_PC	1	OM2_M2_PC_1	5
OM2	M2	MC	OM2_M2	OM2_PC	M2_PC	OM2_M2_PC	2	OM2_M2_PC_2	5
OM2	M2	MC	OM2_M2	OM2_PC	M2_PC	OM2_M2_PC	3	OM2_M2_PC_3	6
OM2	MC	P1	OM2_MC	OM2_P1	MC_P1	OM2_MC_P1	1	OM2_MC_P1_1	4
OM2	MC	P1	OM2_MC	OM2_P1	MC_P1	OM2_MC_P1	2	OM2_MC_P1_2	6
OM2	MC	P1	OM2_MC	OM2_P1	MC_P1	OM2_MC_P1	3	OM2_MC_P1_3	5
OM2	MC	P2	OM2_MC	OM2_P2	MC_P2	OM2_MC_P2	1	OM2_MC_P2_1	6
OM2	MC	P2	OM2_MC	OM2_P2	MC_P2	OM2_MC_P2	2	OM2_MC_P2_2	5
OM2	MC	P2	OM2_MC	OM2_P2	MC_P2	OM2_MC_P2	3	OM2_MC_P2_3	5
OM2	MC	MC	OM2_MC	OM2_PC	MC_PC	OM2_MC_PC	1	OM2_MC_PC_1	5
OM2	MC	MC	OM2_MC	OM2_PC	MC_PC	OM2_MC_PC	2	OM2_MC_PC_2	4
OM2	MC	MC	OM2_MC	OM2_PC	MC_PC	OM2_MC_PC	3	OM2_MC_PC_3	5
T8	M1	P1	T8_M1	T8_P1	M1_P1	T8_M1_P1	1	T8_M1_P1_1	4
T8	M1	P1	T8_M1	T8_P1	M1_P1	T8_M1_P1	2	T8_M1_P1_2	4
T8	M1	P1	T8_M1	T8_P1	M1_P1	T8_M1_P1	3	T8_M1_P1_3	3
T8	M1	P2	T8_M1	T8_P2	M1_P2	T8_M1_P2	1	T8_M1_P2_1	5
T8	M1	P2	T8_M1	T8_P2	M1_P2	T8_M1_P2	2	T8_M1_P2_2	4
T8	M1	P2	T8_M1	T8_P2	M1_P2	T8_M1_P2	3	T8_M1_P2_3	3
T8	M1	MC	T8_M1	T8_PC	M1_PC	T8_M1_PC	1	T8_M1_PC_1	6
T8	M1	MC	T8_M1	T8_PC	M1_PC	T8_M1_PC	2	T8_M1_PC_2	5

T8	M1	MC	T8_M1	T8_PC	M1_PC	T8_M1_PC	3	T8_M1_PC_3	3
T8	M2	P1	T8_M2	T8_P1	M2_P1	T8_M2_P1	1	T8_M2_P1_1	3
T8	M2	P1	T8_M2	T8_P1	M2_P1	T8_M2_P1	2	T8_M2_P1_2	5
T8	M2	P1	T8_M2	T8_P1	M2_P1	T8_M2_P1	3	T8_M2_P1_3	5
T8	M2	P2	T8_M2	T8_P2	M2_P2	T8_M2_P2	1	T8_M2_P2_1	3
T8	M2	P2	T8_M2	T8_P2	M2_P2	T8_M2_P2	2	T8_M2_P2_2	1
T8	M2	P2	T8_M2	T8_P2	M2_P2	T8_M2_P2	3	T8_M2_P2_3	6
T8	M2	MC	T8_M2	T8_PC	M2_PC	T8_M2_PC	1	T8_M2_PC_1	4
T8	M2	MC	T8_M2	T8_PC	M2_PC	T8_M2_PC	2	T8_M2_PC_2	3
T8	M2	MC	T8_M2	T8_PC	M2_PC	T8_M2_PC	3	T8_M2_PC_3	5
T8	MC	P1	T8_MC	T8_P1	MC_P1	T8_MC_P1	1	T8_MC_P1_1	5
T8	MC	P1	T8_MC	T8_P1	MC_P1	T8_MC_P1	2	T8_MC_P1_2	2
T8	MC	P1	T8_MC	T8_P1	MC_P1	T8_MC_P1	3	T8_MC_P1_3	4
T8	MC	P2	T8_MC	T8_P2	MC_P2	T8_MC_P2	1	T8_MC_P2_1	3
T8	MC	P2	T8_MC	T8_P2	MC_P2	T8_MC_P2	2	T8_MC_P2_2	4
T8	MC	P2	T8_MC	T8_P2	MC_P2	T8_MC_P2	3	T8_MC_P2_3	4
T8	MC	MC	T8_MC	T8_PC	MC_PC	T8_MC_PC	1	T8_MC_PC_1	4
T8	MC	MC	T8_MC	T8_PC	MC_PC	T8_MC_PC	2	T8_MC_PC_2	4
T8	MC	MC	T8_MC	T8_PC	MC_PC	T8_MC_PC	3	T8_MC_PC_3	5

Table S2: Overview results for Pearson correlation between the number of guts per sample and OTU richness or Shannon entropy′. Raw and adjusted (adjusted for multiple comparisons through the control of the false discovery rate (FDR)) p-values are given.

		**OTU richness**				**Shannon entropy′**			
	**DF**	**cor**	**t**	**p-value**	**Adjusted p-value**	**cor**	**t**	**p-value**	**Adjusted p-value**
**Genotype**	1	0.917	2.294	0.262	0.366	0.992	7.841	0.081	0.330
**Microbiome**	1	-0.510	-0.593	0.659	0.769	-0.432	-0.478	0.716	0.716

**Parasite**	1	0.957	3.295	0.188	0.328	0.889	1.942	0.303	0.424
**CxM**	7	0.549	1.737	0.126	0.328	0.494	1.504	0.176	0.411
**CxP**	7	0.502	1.537	0.168	0.328	0.590	1.935	0.094	0.330
**MxP**	7	0.055	0.146	0.888	0.888	-0.144	-0.384	0.712	0.716
**CxMxP**	25	0.268	1.391	0.177	0.328	0.211	1.080	0.290	0.424

Table S3: Overview forward and reverse primers used for the internal PCR.

**Name**	**p5-adapter**	**index(8nt)**	**padF(10nt)**	**linkF(2nt)**	**gene-specificprimer**	**Full_primer_sequence**
**Forward**						
F1	AATGATACGGCGACCACCGAGATCTACAC	ATCGTACG	TATGGTAATT	GT	GTGCCAGCMGCCGCGGTAA	AATGATACGGCGACCACCGAGATCTACACATCGTACGTATGGTAATTGTGTGCCAGCMGCCGCGGTAA
F2	AATGATACGGCGACCACCGAGATCTACAC	TAGCGAGT	TATGGTAATT	GT	GTGCCAGCMGCCGCGGTAA	AATGATACGGCGACCACCGAGATCTACACTAGCGAGTTATGGTAATTGTGTGCCAGCMGCCGCGGTAA
F3	AATGATACGGCGACCACCGAGATCTACAC	CGTGAGTG	TATGGTAATT	GT	GTGCCAGCMGCCGCGGTAA	AATGATACGGCGACCACCGAGATCTACACCGTGAGTGTATGGTAATTGTGTGCCAGCMGCCGCGGTAA
F4	AATGATACGGCGACCACCGAGATCTACAC	GATCGTGT	TATGGTAATT	GT	GTGCCAGCMGCCGCGGTAA	AATGATACGGCGACCACCGAGATCTACACGATCGTGTTATGGTAATTGTGTGCCAGCMGCCGCGGTAA
F5	AATGATACGGCGACCACCGAGATCTACAC	ACGACGTG	TATGGTAATT	GT	GTGCCAGCMGCCGCGGTAA	AATGATACGGCGACCACCGAGATCTACACACGACGTGTATGGTAATTGTGTGCCAGCMGCCGCGGTAA
F6	AATGATACGGCGACCACCGAGATCTACAC	ATATACACc	TATGGTAATT	GT	GTGCCAGCMGCCGCGGTAA	AATGATACGGCGACCACCGAGATCTACACATATACACTATGGTAATTGTGTGCCAGCMGCCGCGGTAA
F7	AATGATACGGCGACCACCGAGATCTACAC	GCTCTAGTt	TATGGTAATT	GT	GTGCCAGCMGCCGCGGTAA	AATGATACGGCGACCACCGAGATCTACACGCTCTAGTTATGGTAATTGTGTGCCAGCMGCCGCGGTAA
F8	AATGATACGGCGACCACCGAGATCTACAC	GACactga	TATGGTAATT	GT	GTGCCAGCMGCCGCGGTAA	AATGATACGGCGACCACCGAGATCTACACGACACTGATATGGTAATTGTGTGCCAGCMGCCGCGGTAA
F9	AATGATACGGCGACCACCGAGATCTACAC	AAGCAGCA	TATGGTAATT	GT	GTGCCAGCMGCCGCGGTAA	AATGATACGGCGACCACCGAGATCTACACAAGCAGCATATGGTAATTGTGTGCCAGCMGCCGCGGTAA
F10	AATGATACGGCGACCACCGAGATCTACAC	CGATCTAC	TATGGTAATT	GT	GTGCCAGCMGCCGCGGTAA	AATGATACGGCGACCACCGAGATCTACACCGATCTACTATGGTAATTGTGTGCCAGCMGCCGCGGTAA

F11	AATGATACGGCGACCACCGAGATCTACAC	TGCGTCAC	TATGGTAATT	GT	GTGCCAGCMGCCGCGGTAA	AATGATACGGCGACCACCGAGATCTACACTGCGTCACTATGGTAATTGTGTGCCAGCMGCCGCGGTAA
F12	AATGATACGGCGACCACCGAGATCTACAC	AGAGTCAC	TATGGTAATT	GT	GTGCCAGCMGCCGCGGTAA	AATGATACGGCGACCACCGAGATCTACACAGAGTCACTATGGTAATTGTGTGCCAGCMGCCGCGGTAA
F13	AATGATACGGCGACCACCGAGATCTACAC	ACGCGTGA	TATGGTAATT	GT	GTGCCAGCMGCCGCGGTAA	AATGATACGGCGACCACCGAGATCTACACACGCGTGATATGGTAATTGTGTGCCAGCMGCCGCGGTAA
F14	AATGATACGGCGACCACCGAGATCTACAC	GTCTAGTG	TATGGTAATT	GT	GTGCCAGCMGCCGCGGTAA	AATGATACGGCGACCACCGAGATCTACACGTCTAGTGTATGGTAATTGTGTGCCAGCMGCCGCGGTAA
F15	AATGATACGGCGACCACCGAGATCTACAC	TCTACACT	TATGGTAATT	GT	GTGCCAGCMGCCGCGGTAA	AATGATACGGCGACCACCGAGATCTACACTCTACACTTATGGTAATTGTGTGCCAGCMGCCGCGGTAA
F16	AATGATACGGCGACCACCGAGATCTACAC	GGAGACTA	TATGGTAATT	GT	GTGCCAGCMGCCGCGGTAA	AATGATACGGCGACCACCGAGATCTACACGGAGACTATATGGTAATTGTGTGCCAGCMGCCGCGGTAA
**Reverse**						
R1	CAAGCAGAAGACGGCATACGAGAT	ACTATGTC	AGTCAGTCAG	CC	GGACTACNVGGGTWTCTAAT	CAAGCAGAAGACGGCATACGAGATACTATGTCAGTCAGTCAGCCGGACTACNVGGGTWTCTAAT
R2	CAAGCAGAAGACGGCATACGAGAT	AGTAGCGT	AGTCAGTCAG	CC	GGACTACNVGGGTWTCTAAT	CAAGCAGAAGACGGCATACGAGATAGTAGCGTAGTCAGTCAGCCGGACTACNVGGGTWTCTAAT
R3	CAAGCAGAAGACGGCATACGAGAT	CGTACTCA	AGTCAGTCAG	CC	GGACTACNVGGGTWTCTAAT	CAAGCAGAAGACGGCATACGAGATCGTACTCAAGTCAGTCAGCCGGACTACNVGGGTWTCTAAT
R4	CAAGCAGAAGACGGCATACGAGAT	TAGCAGAC	AGTCAGTCAG	CC	GGACTACNVGGGTWTCTAAT	CAAGCAGAAGACGGCATACGAGATTAGCAGACAGTCAGTCAGCCGGACTACNVGGGTWTCTAAT
R5	CAAGCAGAAGACGGCATACGAGAT	TCATAGAC	AGTCAGTCAG	CC	GGACTACNVGGGTWTCTAAT	CAAGCAGAAGACGGCATACGAGATTCATAGACAGTCAGTCAGCCGGACTACNVGGGTWTCTAAT
R6	CAAGCAGAAGACGGCATACGAGAT	AGCTGCTA	AGTCAGTCAG	CC	GGACTACNVGGGTWTCTAAT	CAAGCAGAAGACGGCATACGAGATAGCTGCTAAGTCAGTCAGCCGGACTACNVGGGTWTCTAAT
R7	CAAGCAGAAGACGGCATACGAGAT	CTCGTTAC	AGTCAGTCAG	CC	GGACTACNVGGGTWTCTAAT	CAAGCAGAAGACGGCATACGAGATCTCGTTACAGTCAGTCAGCCGGACTACNVGGGTWTCTAAT
R8	CAAGCAGAAGACGGCATACGAGAT	GCGCACGT	AGTCAGTCAG	CC	GGACTACNVGGGTWTCTAAT	CAAGCAGAAGACGGCATACGAGATGCGCACGTAGTCAGTCAGCCGGACTACNVGGGTWTCTAAT
R9	CAAGCAGAAGACGGCATACGAGAT	CTAGCTCG	AGTCAGTCAG	CC	GGACTACNVGGGTWTCTAAT	CAAGCAGAAGACGGCATACGAGATCTAGCTCGAGTCAGTCAGCCGGACTACNVGGGTWTCTAAT
R10	CAAGCAGAAGACGGCATACGAGAT	GTATGACG	AGTCAGTCAG	CC	GGACTACNVGGGTWTCTAAT	CAAGCAGAAGACGGCATACGAGATGTATGACGAGTCAGTCAGCCGGACTACNVGGGTWTCTAAT
R11	CAAGCAGAAGACGGCATACGAGAT	TACACAGT	AGTCAGTCAG	CC	GGACTACNVGGGTWTCTAAT	CAAGCAGAAGACGGCATACGAGATTACACAGTAGTCAGTCAGCCGGACTACNVGGGTWTCTAAT

R12	CAAGCAGAAGACGGCATACGAGAT	TCAGCGTT	AGTCAGTCAG	CC	GGACTACNVGGGTWTCTAAT	CAAGCAGAAGACGGCATACGAGATTCAGCGTTAGTCAGTCAGCCGGACTACNVGGGTWTCTAAT

Supplementary information

RESULTS

Table S4: Results of post-hoc analyses on body size, survival, OTU richness, Shannon entropy′ and microbial community composition for the significant results of the statistical analysis (see Table 1). Raw and adjusted (adjusted for multiple comparisons through the control of the false discovery rate (FDR)) p-values are given for the results on *Daphnia* gut microbial communities.

				**Body size**		**Survival**		**OUT richness**			**Shannon entropy′**			**Microbial community composition**		
			**DF**	**p-value**	**z-value**	**p-value**	**z-value**	**p-value**	**Adjusted p-value**	**z-value**	**p-value**	**Adjusted p-value**	**z-value**	**p-value**	**Adjusted p-value**	**R^2**
Genotype		KNO vs OM2	1			**0.001**	3.31	**<0.001**	**0.003**	3.588	**0.002**	**0.007**	3.347	**0.003**	**0.009**	0.069
		KNO vs T8	1			0.634	-0.48	0.275	0.275	1.530	0.111	0.167	1.992	**0.006**	**0.009**	0.073
		OM2 vs T8	1			**<0.001**	-4.54	0.052	0.078	- 2.325	0.195	0.195	-1.718	0.092	0.092	0.035
Microbiome		M1 vs M2	**1**	**0.031**	- 2.529											
		M1 vs MC	1	0.633	- 0.912											
		M2 vs MC	1	0.227	1.654											
Parasite		P1 vs P2	1			0.567	-0.57									
		P1 vs PC	1			**0.007**	3.26									
		P2 vs PC	1			**0.001**	2.61									
Genotype x Microbiome	Within KNO	M1 vs M2	1					1.000	1.000	0.459	0.999	1.000	0.708			
		M1 vs MC	1					1.000	1.000	- 0.018	1.000	1.000	0.375			
		M2 vs MC	1					1.000	1.000	- 0.461	1.000	1.000	-0.337			
	Withi n	M1 vs M2	1					0.997	1.000	- 0.802	0.999	1.000	-0.673			

		M1 vs MC	1				**0.024**	0.142	-3.403	**0.018**	0.164	-3.395	
		M2 vs MC	1				0.142	0.639	-2.703	0.070	0.315	-2.957	
	Within T8	M1 vs M2	1				0.998	1.000	0.731	0.998	1.000	0.720	
		M1 vs MC	1				0.764	1.000	1.660	0.773	1.000	1.632	
		M2 vs MC	1				0.987	1.000	0.985	0.985	1.000	0.995	
	Within M1	KNO vs OM2	1				**0.008**	0.142	3.626	**0.013**	0.164	-3.395	
		KNO vs T8	1				1.000	1.000	0.278	0.993	1.000	0.895	
		OM2 vs T8	1				**0.018**	0.142	-3.403	0.065	0.315	-2.985	
	Within M2	KNO vs OM2	1				0.297	1.000	2.363	0.293	1.000	2.354	
		KNO vs T8	1				1.000	1.000	0.470	0.997	1.000	0.788	
		OM2 vs T8	1				0.375	1.000	-2.235	0.513	1.000	-2.014	
	Within MC	KNO vs OM2	1				1.000	1.000	0.526	1.000	1.000	0.106	
		KNO vs T8	1				0.634	1.000	1.860	0.543	1.000	1.974	
		OM2 vs T8	1				0.860	1.000	1.481	0.471	1.000	2.077	
Genotype x Parasite	Within KNO	P1 vs P2	1		0.894	0.13							
		P1 vs PC	1		**<0.001**	3.52							
		P2 vs PC	1		**<0.001**	3.69							
	Within OM2	P1 vs P2	1		0.134	-1.50							
		P1 vs PC	1		0.699	0.39							
		P2 vs PC	1		0.206	-1.26							

	Within T8	P1 vs P2	1		0.907	0.12					
		P1 vs PC	1		0.232	1.20					
		P2 vs PC	1		0.217	1.24					
	Within P1	KNO vs OM2	1		**0.013**	2.48					
		KNO vs T8	1		0.504	0.67					
		OM2 vs T8	1		**0.023**	-2.28					
	Within P2	KNO vs OM2	1		**<.0001**	4.08					
		KNO vs T8	1		0.484	0.70					
		OM2 vs T8	1		**<0.001**	-3.96					
	Within PC	KNO vs OM2	1		0.255	-1.14					
		KNO vs T8	1		**0.019**	-2.35					
		OM2 vs T8	1		0.116	-1.57					
Microbiome x Parasite	Within M1	P1 vs P2	1					1.000	1.000	0.008	
		P1 vs PC	1					0.898	1.000	1.390	
		P2 vs PC	1					0.970	1.000	1.120	
	Within M2	P1 vs P2	1					1.000	1.000	-0.587	
		P1 vs PC	1					0.664	1.000	-1.815	
		P2 vs PC	1					0.931	1.000	-1.293	

Within MC	P1 vs P2	1				1.000	1.000	0.192	
	P1 vs PC	1				0.979	1.000	1.057	
	P2 vs PC	1				0.992	1.000	0.913	
Within P1	M1 vs M2	1				0.789	1.000	1.618	
	M1 vs MC	1				1.000	1.000	-0.368	
	M2 vs MC	1				0.635	1.000	-1.856	
Within P2	M1 vs M2	1				0.995	1.000	0.855	
	M1 vs MC	1				1.000	1.000	-0.163	
	M2 vs MC	1				0.951	1.000	-1.214	
Within PC	M1 vs M2	1		-		0.794	1.000	-1.609	
	M1 vs MC	1				0.999	1.000	-0.639	
	M2 vs MC	1				0.984	1.000	1.009	

Table S5: Overview of relative abundances of the 40 most common OTUs in *Daphnia* guts from the microbiome transplant experiment. Abundances were calculated on rarefied data. Sd: standard deviation.

**Class**	**Order**	**Family**	***Genus***	**OTU**	**Mean**	**Sd**
Gammaproteobacteria	Betaproteobacteriales	Burkholderiaceae	*NA*	OTU_1_*Burkholderiaceae*	29.94%	28.01%
Alphaproteobacteria	Rhizobiales	Beijerinckiaceae	*Methylobacterium*	OTU_2_*Methylobacterium*_sp.	6.59%	15.64%
Bacilli	Lactobacillales	Streptococcaceae	*Streptococcus*	OTU_4_*Streptococcus*_sp.	6.09%	13.60%
Gammaproteobacteria	Pseudomonadales	Moraxellaceae	*Acinetobacter*	OTU_3_*Acinetobacter*_sp.	5.43%	14.72%
Alphaproteobacteria	Rickettsiales	Rickettsiales_Incertae_Sedis	*Candidatus_Hepatincola*	OTU_6_*Candidatus_Hepatincola*_sp.	3.48%	6.23%
Gammaproteobacteria	Betaproteobacteriales	Burkholderiaceae	*Hydrogenophaga*	OTU_9_*Hydrogenophaga*_sp.	2.63%	5.56%
Alphaproteobacteria	Rhizobiales	Rhizobiaceae	*Shinella*	OTU_5_*Shinella*_sp.	2.60%	9.35%
Gammaproteobacteria	Pseudomonadales	Moraxellaceae	*Acinetobacter*	OTU_7_*Acinetobacter*_sp.	2.25%	5.68%

Bacteroidia	Flavobacteriales	Flavobacteriaceae	*Flavobacterium*	OTU_10_*Flavobacterium*_sp.	1.97%	6.65%
Actinobacteria	Micrococcales	Microbacteriaceae	*Candidatus_Limnoluna*	OTU_13_*Candidatus_Limnoluna*_sp.	1.89%	3.04%
Bacteroidia	*NA*	*NA*	*NA*	OTU_12_Bacteroidia	1.76%	3.55%
Gammaproteobacteria	Betaproteobacteriales	T34	*NA*	OTU_11_Betaproteobacteriales	1.65%	5.84%
Gammaproteobacteria	Enterobacteriales	Enterobacteriaceae	*Escherichia/Shigella*	OTU_15_*Escherichia/Shigella*_sp.	1.53%	2.67%
Actinobacteria	Micrococcales	Microbacteriaceae	*Candidatus_Planktoluna*	OTU_19_*Candidatus_Planktoluna*_sp.	1.28%	2.20%
Gammaproteobacteria	Betaproteobacteriales	Burkholderiaceae	*NA*	OTU_8_Burkholderiaceae	1.26%	1.67%
Bacteroidia	Cytophagales	Cyclobacteriaceae	*Algoriphagus*	OTU_14_*Algoriphagus*_sp.	1.14%	2.60%
Gammaproteobacteria	Betaproteobacteriales	Burkholderiaceae	*Polynucleobacter*	OTU_22_*Polynucleobacter*_sp.	1.13%	1.91%
Gammaproteobacteria	Betaproteobacteriales	Burkholderiaceae	*Rhodoferax*	OTU_16_*Rhodoferax*_sp.	1.06%	2.66%
Gammaproteobacteria	Betaproteobacteriales	Burkholderiaceae	*NA*	OTU_18_Burkholderiaceae	0.86%	1.97%
Bacteroidia	Flavobacteriales	Weeksellaceae	*Empedobacter*	OTU_24_*Empedobacter*_sp.	0.86%	1.35%
Gammaproteobacteria	Betaproteobacteriales	Burkholderiaceae	*Polynucleobacter*	OTU_27_*Polynucleobacter*_sp.	0.79%	4.82%
Gammaproteobacteria	Betaproteobacteriales	Burkholderiaceae	*NA*	OTU_30_Burkholderiaceae	0.79%	3.00%
Gammaproteobacteria	Betaproteobacteriales	Burkholderiaceae	*Burkholderia-Caballeronia-Paraburkholderia*	OTU_34_*Burkholderia-Caballeronia-Paraburkholderia*_sp.	0.74%	1.87%
Gammaproteobacteria	Betaproteobacteriales	Burkholderiaceae	*NA*	OTU_21_Burkholderiaceae	0.67%	1.42%
Gammaproteobacteria	Betaproteobacteriales	T34	*NA*	OTU_17_Betaproteobacteriales	0.64%	2.81%
Bacteroidia	Flavobacteriales	Flavobacteriaceae	*Flavobacterium*	OTU_26_*Flavobacterium*_sp.	0.62%	2.50%
Gammaproteobacteria	Betaproteobacteriales	T34	*NA*	OTU_20_Betaproteobacteriales	0.53%	2.37%
Gammaproteobacteria	Pseudomonadales	Moraxellaceae	*Acinetobacter*	OTU_38_*Acinetobacter*_sp.	0.53%	1.22%
Bacteroidia	Flavobacteriales	Flavobacteriaceae	*Flavobacterium*	OTU_23_*Flavobacterium*_sp.	0.46%	3.95%
Gammaproteobacteria	Betaproteobacteriales	Burkholderiaceae	*Polynucleobacter*	OTU_39_*Polynucleobacter*_sp.	0.46%	0.85%
Mollicutes	Mycoplasmatales	Mycoplasmataceae	*Candidatus_Bacilloplasma*	OTU_50_*Candidatus_Bacilloplasma*_sp.	0.46%	3.47%
Gammaproteobacteria	Betaproteobacteriales	Burkholderiaceae	*NA*	OTU_45_Burkholderiaceae	0.45%	1.00%
Gammaproteobacteria	Betaproteobacteriales	*NA*	*NA*	OTU_54_Betaproteobacteriales	0.41%	2.95%
Gammaproteobacteria	Betaproteobacteriales	Burkholderiaceae	*Massilia*	OTU_25_*Massilia*_sp.	0.40%	3.45%
Actinobacteria	Micrococcales	Microbacteriaceae	*Aurantimicrobium*	OTU_41_*Aurantimicrobium*_sp.	0.38%	0.51%
Bacilli	Bacillales	Staphylococcaceae	*Staphylococcus*	OTU_40_*Staphylococcus*_sp.	0.36%	1.01%
Gammaproteobacteria	Pseudomonadales	Moraxellaceae	*Acinetobacter*	OTU_29_*Acinetobacter*_sp.	0.35%	1.09%

Gammaproteobacteria	Betaproteobacteriales	Burkholderiaceae	*NA*	OTU_35_Burkholderiaceae	0.33%	0.72%
Gammaproteobacteria	Pseudomonadales	Moraxellaceae	*Acinetobacter*	OTU_28_*Acinetobacter*_sp.	0.33%	1.02%
Bacilli	Bacillales	Family_XI	*Gemella*	OTU_47_*Gemella*_sp.	0.30%	2.03%

Table S6: Results Deseq analysis on class level and OTU level between main effects.

	Genotype			Microbiome			Parasite		
	**KNO VS OM2**	**KNO VS T8**	**OM2 VS T8**	**M1 vs M2**	**M2 vs MC**	**M1 vs MC**	**P1 vs P2**	**P2 vs PC**	**P1 vs PC**
**Class level**									
**Gammaproteobacteria**	0.001				<0.001				
**Alphaproteobacteria**	0.002	0.003	<0.001		<0.001	<0.001			<0.001
**Bacilli**				0.012	0.010	0.003	0.034		
**Bacteroidia**	0.002	0.004			<0.001	<0.001			
**Actinobacteria**				0.025					
**OTU level**									
**OTU_2_Methylobacterium_sp.**	<0.001		<0.001	0.003	<0.001	0.001		0.005	<0.001
**OTU_3_Acinetobacter_sp.**	<0.001	0.014					<0.001	0.003	
**OTU_4_Streptococcus_sp.**			0.006	0.013		<0.001			
**OTU_5_Shinella_sp.**					0.036	<0.001			
**OTU_6_Candidatus_Hepatincola_sp.**		0.018	0.002	0.010		<0.001			
**OTU_7_Acinetobacter_sp.**	<0.001	<0.001		0.026			<0.001	0.001	
**OTU_10_Flavobacterium_sp.**	0.048	0.002							
**OTU_11_Betaproteobacteriales**	<0.001	<0.001				0.007			
**OTU_12_Bacteroidia**	<0.001	<0.001							
**OTU_13_Candidatus_Limnoluna_sp.**		<0.001	0.002			0.001			
**OTU_14_Algoriphagus_sp.**	<0.001	<0.001							
**OTU_15_Escherichia/Shigella_sp.**				0.031		0.012			
**OTU_16_Rhodoferax_sp.**	<0.001	<0.001		0.021		0.001		0.027	
**OTU_17_Betaproteobacteriales**				0.010		<0.001			
**OTU_18_Burkholderiaceae**			0.005			0.026			
**OTU_19_Candidatus_Planktoluna_sp.**	0.006	<0.001	0.001						
**OTU_20_Betaproteobacteriales**				0.012		<0.001			
**OTU_22_Polynucleobacter_sp.**	<0.001		<0.001						

**OTU_24_Empedobacter_sp.**	<0.001	<0.001	0.001						
**OTU_26_Flavobacterium_sp.**			0.015						
**OTU_27_Polynucleobacter_sp.**		<0.001	<0.001			0.009			
**OTU_28_Acinetobacter_sp.**	<0.001	<0.001					<0.001		<0.001
**OTU_29_Acinetobacter_sp.**							<0.001		0.022
**OTU_31_Betaproteobacteriales**						<0.001			
**OTU_34_Burkholderia-Caballeronia-Paraburkholderia_sp.**	<0.001	<0.001							
**OTU_35_Burkholderiaceae**			0.019						
**OTU_37_Pseudorhodobacter_sp.**	0.011						<0.001		<0.001
**OTU_38_Acinetobacter_sp.**	<0.001								
**OTU_39_Polynucleobacter_sp.**		<0.001	<0.001						
**OTU_40_Staphylococcus_sp.**				<0.001					
**OTU_41_Aurantimicrobium_sp.**	<0.001	<0.001		0.008		0.010		0.034	
**OTU_42_Rhodoferax_sp.**	0.005	<0.001							
**OTU_43_Shinella_sp.**						0.026	<0.001	0.045	
**OTU_45_Burkholderiaceae**	<0.001	<0.001	0.018						
**OTU_46_Acinetobacter_sp.**		0.018							<0.001
**OTU_47_Gemella_sp.**				0.009	0.038				
**OTU_48_Streptococcus_sp.**	0.040								
**OTU_50_Candidatus_Bacilloplasma_sp.**	0.017			0.002					
**OTU_52_Acinetobacter_sp.**							<0.001		<0.001
**OTU_53_Aeromonas_sp.**	0.047								
**OTU_54_Betaproteobacteriales**				0.050	0.024				
**OTU_56_Streptococcus_sp.**	<0.001					0.034			
**OTU_57_Limnohabitans_sp.**		0.003							
**OTU_59_Burkholderiaceae**		<0.001	<0.001						
**OTU_60_GKS98_freshwater_group_sp.**			0.003						
**OTU_61_Cutibacterium_sp.**			0.029						
**OTU_62_Pedobacter_sp.**							<0.001	0.034	
**OTU_63_Pseudomonas_sp.**		0.007							
**OTU_65_Prevotella_sp.**	0.005		0.046				0.022	0.045	
**OTU_66_Bradyrhizobium_sp.**	<0.001								
**OTU_67_Aurantimicrobium_sp.**		0.033		0.010			<0.001		<0.001
**OTU_69_Prevotella_7_sp.**	<0.001		0.006				0.002	0.007	

**OTU_70_Sphingomonas_sp.**	<0.001								
**OTU_72_Limnohabitans_sp.**	0.004		0.018						
**OTU_74_Rothia_sp.**	0.005						0.009		
**OTU_75_Haemophilus_sp.**	<0.001						0.020		<0.001
**OTU_76_Gemmobacter_sp.**	0.038						0.004	0.027	
**OTU_77_Burkholderiaceae**			0.042						
**OTU_78_Cutibacterium_sp.**						0.023			
**OTU_79_Neisseria_sp.**	0.038					0.003			
**OTU_83_Gemmobacter_sp.**	<0.001						<0.001		<0.001
**OTU_87_Flavobacterium_sp.**	0.015					0.019			
**OTU_88_Legionella_sp.**						0.029			
**OTU_90_Veillonella_sp.**	<0.001		0.007				0.004	0.044	
**OTU_91_Streptococcus_sp.**					0.036				
**OTU_92_Paenibacillus_sp.**		0.014							
**OTU_100_Shinella_sp.**	<0.001					<0.001			
**OTU_102_Shinella_sp.**						<0.001	0.032		

Table S7. Significant results of the statistical analysis on the effect of genotype, microbiome treatment, parasite community treatment and their interactions on body size, survival and alpha-diversity variables (OTU richness, Shannon entropy'). Obtained P-values were adjusted for multiple comparisons through the control of the false discovery rate (FDR). Significant data ($p < 0.05$) is indicated in bold. Highly significant data ($p < 0.001$) are underlined. Raw and adjusted (adjusted for multiple comparisons through the control of the false discovery rate (FDR)) p-values are given for the results on *Daphnia* gut microbial communities.

	DF	Body size	Survival	OTU richness		Shannon entropy'		Microbial community composition		
		p-value	p-value	p-value	Adjusted p-value	p-value	Adjusted p-value	R^2	p-value	Adjusted p-value
Genotype	2	0.857	**<u><0.001</u>**	**<u><0.001</u>**	**<u><0.001</u>**	**<u><0.001</u>**	**<u><0.001</u>**	0.075	**0.002**	**0.014**

Microbiome treatment	2	**0.034**		0.279	0.348	0.843	0.859	0.030	0.296	0.414
Parasite treatment	2	0.334	**0.002**	0.298	0.348	0.216	0.379	0.029	0.266	0.414
Genotype x Microbiome treatment	4	0.240		**0.0043**	**0.015**	**0.001**	**0.004**	0.066	0.146	0.414
Genotype x Parasite treatment	4	0.086	**0.003**	0.4831	0.483	0.690	0.859	0.048	0.539	0.539
Microbiome treatment x Parasite treatment	4	0.079		0.2080	0.348	**0.020**	**0.047**	0.053	0.430	0.502
Genotype x Microbiome treatment x Parasite treatment	8	0.478		0.2461	0.348	0.859	0.859	0.122	0.197	0.414

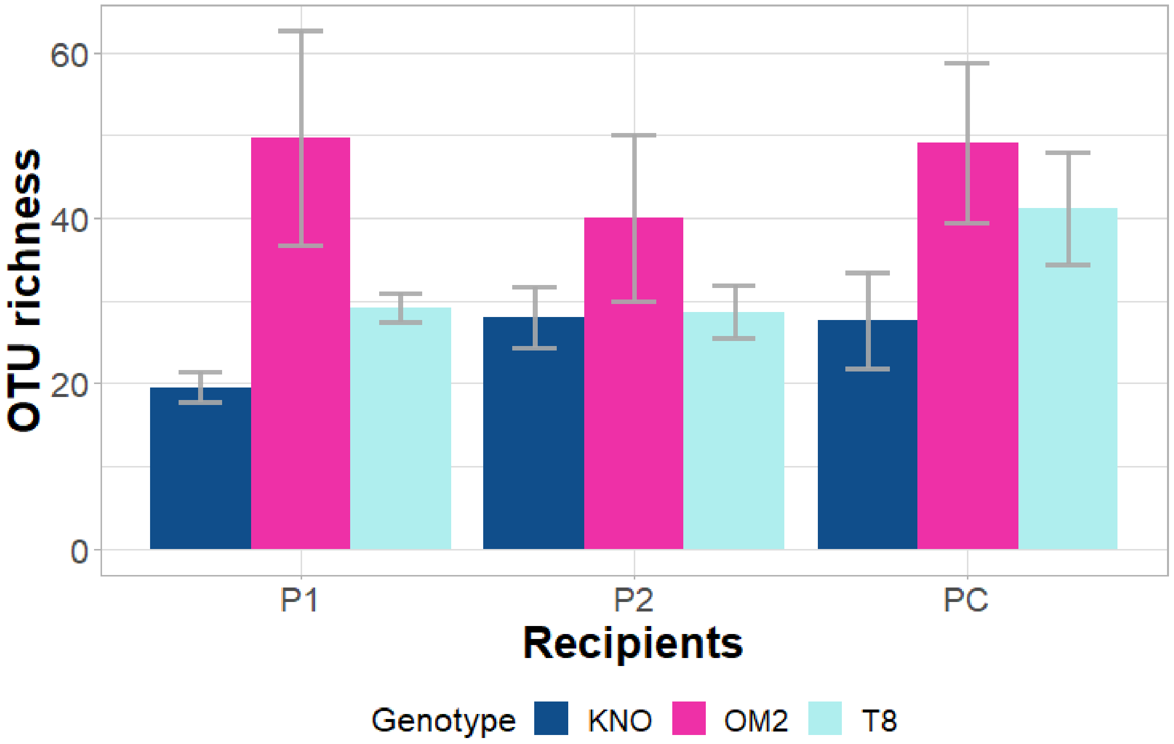

Figure S1. Effect of the genotype x parasite interaction on OTU richness. Colours indicate the different genotypes. Error bars indicate standard error.

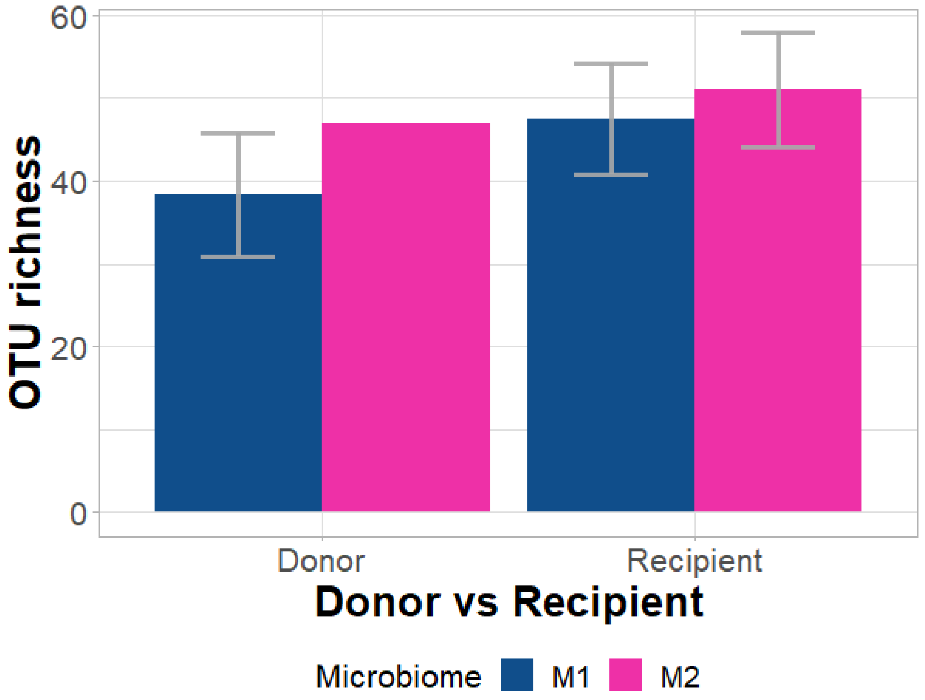

Figure S2: Effect of microbiome (M1 and M2) for donor and recipient samples on OTU richness. Error bars indicate standard error. Colours indicate the different microbiome treatments. OTU richness in the M1 inoculum (mean=38.333, sd=13.051) was, on average, lower compared with the M2 inoculum

(OTU richness=47.000).,

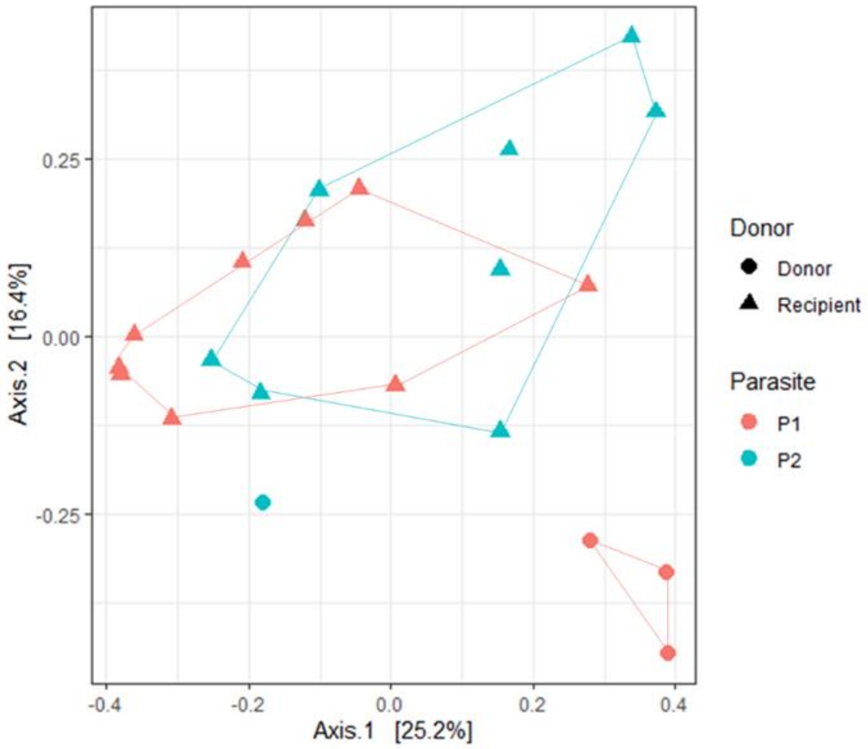

Figure S3: PCA of the gut microbial communities of recipients using weighted UniFrac distance for the donors (P1 and P2) and matching recipients (M1 x P1, M2 x P2) using weighted UniFrac distance for donor/recipient type and parasite treatment. Analyses on donor (P1 and P2) and matching recipient (M1 x P1, M2 x P2) bacterial communities, showed a significant difference in structure for P1 ($p=0.005$; $R2=0.147$), but not for P2 ($p=0.783$; $R2=0.330$). Bray-Curtis ordinations revealed that both P1 and P2, showed complete segregation between donors and recipients (Figure 7b), indicating that the donors and recipients for both the P1 and P2 treatment were differently structured (Figure 7b), however non-significant for P2.
